# Dietary riboflavin (vitamin B2) intake and osteoporosis in U.S. female adults: unveiling of association and exploration of potential molecular mechanisms

**DOI:** 10.1186/s12937-025-01103-x

**Published:** 2025-04-07

**Authors:** QianKun Yang, Li Zhang, Dong Sun, Shen Jie, XiaoLiang Tao, Qing Meng, Fei Luo

**Affiliations:** 1 National & Regional United Engineering Lab of Tissue Engineering, Department of Orthopedics, Southwest Hospital, Army Medical University, Chongqing, 400038 China; 2 Department of Hematology, National Clinical Research Center for Child Health and Disorders, Ministry of Education Key Laboratory of Child Development and Disorders, Chongqing Key Laboratory of Pediatrics, Children’s Hospital of Chongqing Medical University, No.136 of Zhong Shan Second Road, YuZhong District, Chongqing, 400014 China; 3 Department of Neurology, National Clinical Research Center for Child Health and Disorders, Ministry of Education Key Laboratory of Child Development and Disorders, Chongqing Key Laboratory of Pediatrics, Children’s Hospital of Chongqing Medical University, No.136 of Zhong Shan Second Road, YuZhong District, Chongqing, 400014 China; 4 Department of Orthopedic Surgery, Beijing Jishuitan Hospital Guizhou Hospital, Guiyang, 550000 China; 5 Department of Orthopaedics, Southwest Hospital, Third Military Medical University (Army Medical University), No.29 Gaotanyan St., Shapingba District, Chongqing, 400038 China

**Keywords:** Riboflavin, Vitamin B2, Osteoporosis, Bone mineral density

## Abstract

**Background:**

Osteoporosis characterized by deteriorating bone loss is becoming one of the serious health problems globally. Vitamin B2, also known as riboflavin, exhibiting multiple prominent physiological traits such as antioxidant effects, reducing lipid peroxidation and regulating glutathione redox cycle, allows it to be a potential agent to improve bone loss. However, the relationship between dietary vitamin B2 intake and osteoporosis remains unelucidated. The objective of this study was to explore the association between the dietary intake of vitamin B2 and bone loss in the U.S. female adults using the National Health and Nutrition Examination Survey (NHANES) database.

**Methods:**

Female participants with complete information on dietary vitamin B2 intake, dual-energy X-ray absorptiometry, and other essential covariates from NHANES database were included in the current study. Multivariable logistic regression and linear regression analyses were conducted to assess the relationships of dietary vitamin B2 intake with osteoporosis and bone mineral density (BMD) levels, respectively. Subgroup analyses, interaction tests, and restricted cubic spline (RCS) regression analyses were further used to verify the stability, robustness and potential nonlinearity of the association. Mediation analysis was performed to probe the role of serum alkaline phosphatase (ALP) in the aforementioned relationship, and the network pharmacology analysis was also conducted to determine the potential pathways and key targets for vitamin B2 regulating bone health.

**Results:**

A total of 4, 241 female participants from four NHANES cycles were included in this study. After multivariate adjustment, the intake of vitamin B2 was beneficially associated with reduced risk for femur osteoporosis (OR_Q4 vs. Q1_=0.613; 95%CI: 0.454–0.829). A higher intake of vitamin B2 (quartile 4) was significantly correlated with decreased risk of reduced femoral BMD levels, with the β being 0.020 (95%CI: 0.007–0.033), 0.015 (95%CI: 0.002–0.027), 0.020 (95%CI: 0.009–0.031) and 0.022 (95%CI: 0.006–0.037) for the BMD of total femur, femoral neck, trochanter, and intertrochanter, respectively (all P value < 0.05). Covariate total MET was found to modify the association between vitamin B2 intake and osteoporosis (P interaction = 0.0364), with the aforementioned relationship being more pronounced in the subgroup of insufficiently active individuals. Furthermore, RCS analysis revealed that vitamin B2 intake was positively and linearly associated with reduced risk for femoral OP and increased BMD levels of total femur, trochanter and intertrochanter, while positively and nonlinearly correlated with increased BMD level of femoral neck. Additionally, the association between vitamin B2 intake, osteoporosis and BMD levels was mediated by ALP, with a mediation proportion of 12.43%, 7.58%, 12.17%, 7.64%, and 6.99% for OP, total femur, femoral neck, trochanter, and intertrochanter BMD, respectively. Finally, network pharmacology analysis indicated that vitamin B2 regulating bone health mainly through pathways like HIF-1 signaling pathway, longevity regulating pathway, p53 signaling pathway, etc.

**Conclusions:**

Higher intake of vitamin B2 is positively associated with reduced risks for femoral osteoporosis and bone loss. Vitamin B2 may represent a modifiable lifestyle factor for the prevention and management of osteoporosis.

**Supplementary Information:**

The online version contains supplementary material available at 10.1186/s12937-025-01103-x.

## Introduction

Osteoporosis is a systemic skeletal disorder characterized by low bone mass and damage to the bone microstructure, resulting in continuous bone loss and increased bone fragility, as well as susceptibility to fragile fracture [1]. Osteoporosis is called a silent epidemic for it usually goes undiagnosed until fragile fractures occur, leading to severe complications, such as chronic pain, disability and even death [2, 3]. In China, a cross-sectional study included 20, 416 individuals from 2017 to 2018 revealed that the weighted prevalence of osteoporosis in adults over 40 years old was 5.0% among men and 20.6% among women, while the prevalence vertebral fracture was 10.5% and 9.7% for men and women [4], respectively. Just in the United States, there was more than 54 million older adults suffer osteoporosis, contributing to heavy medical burden [5]. Moreover, it is estimated that almost 2–7 million hip fractures occurred globally in 2010, with 51% of which can be avoided if the osteoporosis was effectively prevented [6, 7]. Therefore, the prevention and management of osteoporosis has become a crucial public health issue currently.

Women and men exhibited obviously distinct characteristics in bone loss during aging process. Usually, both women and men obtained maximum bone mineral density (BMD) (also termed peak BMD) at the age of 25–30 years [8, 9]. After the age of 30 years old, the balance of bone homeostasis tilted from a balanced situation towards bone resorption with an average of 1% bone loss per year, which was independent of gender [8]. Moreover, trabecular bone density measurement showed that BMD gradually decreased from 20 to 80 years of age, and it decreased by about 50% at the age of 80 years, a process determined by genetic program [8]. In postmenopausal women, depletion of estrogen is accompanied by about 4% of bone loss per year, which means that women may lose 40% of their bone mass between the ages of 40 and 70 years as opposed to that of 12% in men [8]. Briefly, from the achievement of peak BMD onwards, men experienced bone loss in a continuous and gradual manner (low bone turnover), while women lose bone mass in a more complexed way, sequentially began from low bone turnover, and then turned into high bone turnover (50–65 years) and finally became low bone turnover (> 65 years) again. Given this, the prevention and management of osteoporosis in female individuals are more complexed and thus need more profound exploration.

As a chronic disease, osteoporosis requires a long-term or lifelong management. Currently, lifestyle interventions, anti-resorptive agents (e.g., bisphosphonates), anabolic drugs (e.g., parathyroid hormone analogs), traditional Chinese medicine (e.g., epimedium total flavone capsule), calcium and vitamin D supplement, etc., constitute the main pillars for the treatment of osteoporosis [1, 8]. However, potential adverse drug effect, fragmented healthcare system and heavy economic burden all pose great challenges to patients’ long-term compliance, which largely weakens the effectiveness of drug therapy. In contrast, dietary interventions as an anti-osteoporotic strategy may represent a more feasible and easy-to-implement approach to alleviate and improve osteoporosis.

Vitamin B2, also known as riboflavin, is abundant in egg yolk, dark green leafy vegetables, grains, beans, nuts, fish, meat and offal, which is an important micronutrient and the direct precursor of flavin mononucleotide (FMN) and flavin adenine dinucleotide (FAD). It plays a pivotal role in the biochemical reactions of all living cells by involving in the metabolism of carbohydrates, lipids, ketone bodies and proteins. Moreover, its potent antioxidant, anti-inflammatory, metabolic regulation, and osteogenic properties confer substantial potential for the enhancement and improvement of bone health. Previous studies have revealed that vitamin B2 can regulate bone metabolism in multiple ways. Firstly, vitamin B2 utilization has been reported to reduce the production or expression of pro-inflammatory cytokines, nitric oxide and COX2 by regulating the NF-κB pathway via its proteasome inhibitory action, leading to the alleviation of inflammation and enhancement of osteogenesis [10, 11]. Secondly, several studies have demonstrated that vitamin B2 can promote the self-renewal and osteogenic differentiation capacities of mesenchymal stem cells via regulating multiple signal pathways, such as p38 MAPK/BMP-2/Smad1/5/9 signaling pathway [12], AKT/FAK/CaMKII pathway [13], and caspases-3/8/9 pathway [13]. Additionally, vitamin B2 has been reported to up-regulate the expression of some osteoblastic transcription factors, such as Runx2 [13], β-catenin [13], and so on. In summary, vitamin B2 may influence bone health at least via improving oxidative stress, enhancing osteogenesis, and promoting osteogenic gene expression. As a result, vitamin B2 may represent a potential and controllable dietary treatment option for the prevention and management of osteoporosis. Currently, it is unfortunately that the existing studies regarding vitamin B2 and bone health mainly focus on in the field of basic research, while clinical studies based on large population to explore the relationship between dietary vitamin B2 intake and bone loss are limited.

Therefore, in this study, we aimed to assess the relationship between dietary intake of riboflavin (vitamin B2) and osteoporosis in female U.S. adult population based on the nationally representative data. Our results are intended to provide important dietary advice and an underlying theoretical justification for the prevention of osteoporosis.

## Materials and methods

### Study population

The National Health and Nutrition Examination Survey (NHANES) is a continuous cross-sectional study conducted by the Centers for Disease Control and Prevention (CDC) aimed at evaluating the health and nutritional status of the US population. NHANES was conducted based on a complex, multistage, stratified, and clustered probability design and updated periodically in 2-year cycles since 1999, within each cycle the data of a representative sample of the US population was collected [14]. Participants’ data from NHANES database mainly included individuals’ demographics, dietary information, examination data, laboratory and other information such as lifestyles, health status, and so on.

**Fig. 1:**
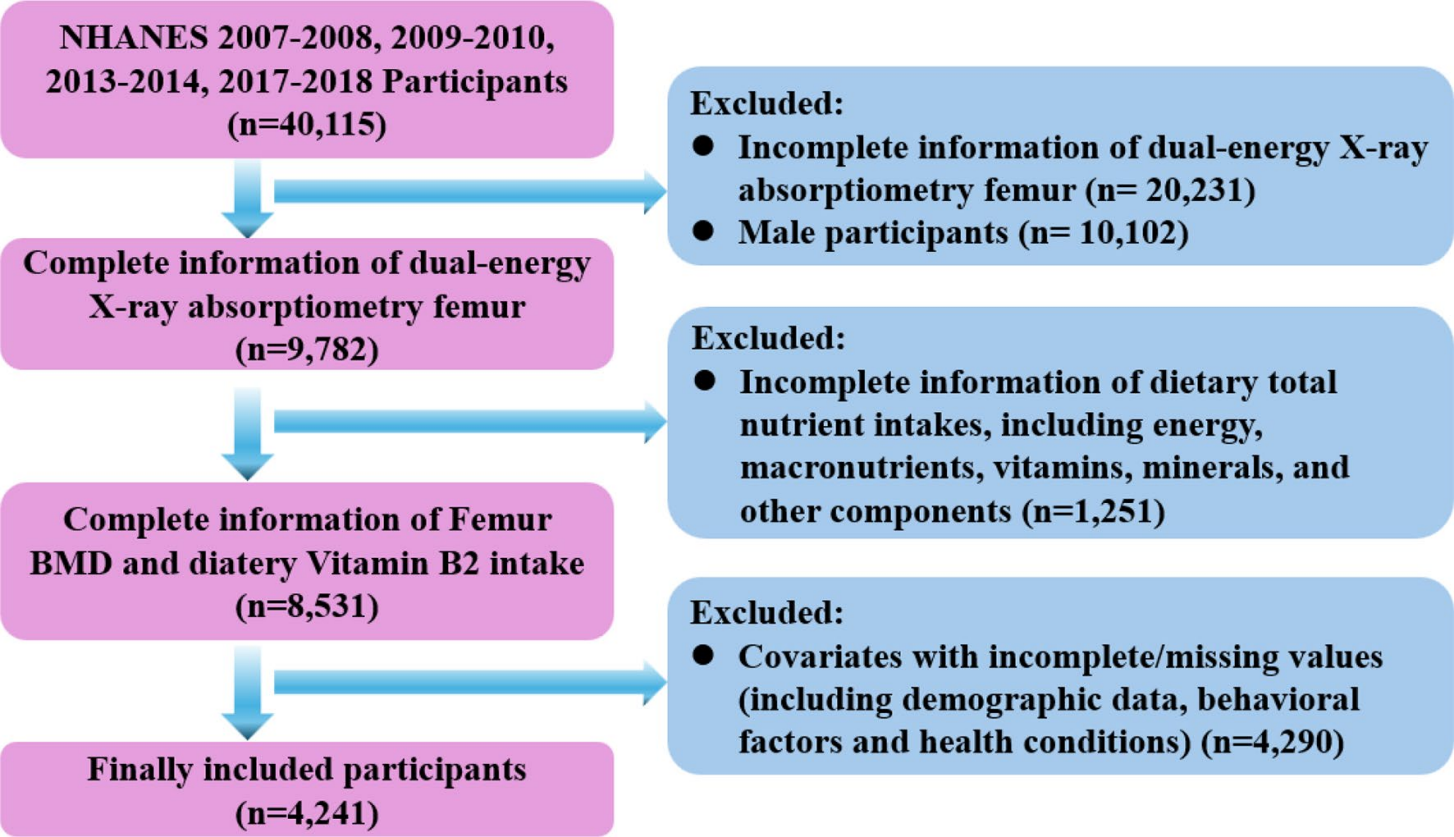
Flow diagram of participant selection

The current study includes participants from NHANSE 2007–2010, 2013–2014 and 2017–2018 whose femur BMD levels were reported, including a total of 40,115 individuals. Individuals with incomplete data on femur dual-energy X-ray absorptiometry, dietary vitamin B2 intake, and other essential covariates were excluded from the analysis. Ultimately, a total of 4241 female participants were included. The flowchart for participant selection was presented in Fig. 1. The program was approved by the National Center for Health Statistics Ethics Review Board. All of the participants provided written informed consent. Thus, no additional ethical review board approval was required to analyze the anonymized NHANES data.

### Bone mineral density, osteoporosis and osteoporotic fractures assessment

The BMD levels at different femur regions (including total femur, femur neck, trochanter, and intertrochanter) were evaluated in the NHANES. Specifically, the Dual-energy X-ray absorptiometry (DXA), Hologic QDR-4500 A fan-beam densitometers (Hologic, Inc., Bedford, Massachusetts) were utilized for examination due in part to its speed, ease of use, and low radiation exposure [15–17].

According to the criteria established by the World Health Organization, the diagnosis of osteoporosis was based on the internationally recognized T-score, which can be calculated using the formula: T-score=(BMD_respondent_-Mean BMD_reference_)/SD_reference_ [7]. The mean BMD reference value for calculating T-score was that of the non-Hispanic white women aged 20–29 from the NHANES III report [18]. Participants with T-score being ≤-2.5, -2.5~-1.0, and ≥-1.0 were defined as osteoporosis, osteopenia and normal, respectively [7]. Thus, the thresholds for the definition of femur osteoporosis in female participants were 0.635 g/cm^2^ for total femur, 0.560 g/cm^2^ for femur neck, 0.4625 g/cm^2^ for trochanter, 0.735 g/cm^2^ for intertrochanter (Table. S1), respectively. In this study, participants’ T-score in any femur regions lower than − 2.5 were defined as osteoporosis, otherwise regarded as non-osteoporosis.

For the assessment of osteoporotic fractures, participants were asked the question of “Has a doctor ever told you that you had broken or fractured hip?”, and those who answered “yes” were recorded as osteoporotic fractures [19].

### Dietary riboflavin (vitamin B2) intake

The evaluation of dietary intake data was based on two 24-hour dietary recall interviews, which was used to estimate intakes of energy, nutrients, and other food components consumed during the 24-hour period prior to the interview. The first one was collected in-person in the Mobile Examination Center (MEC) while the second one was collected by telephone 3–10 days later. A comprehensive outline of the interview procedures is available in the dietary interview section on the NHANES website [20]. The dietary intake levels of riboflavin (vitamin B2) were estimated using the averaged values from the two 24-h recalls of total nutrient intakes, which had considered the complex survey design and sampling methods to ensure representation of the U.S. population aged 18 and older [21]. According to the dietary recall data, average daily intake (mg/day) of riboflavin (vitamin B2) was calculated based on the U.S. Department of Agriculture’s (USDA) Dietary Study Food and Nutrition Database for Dietary Studies (FNDDS) [7].

### Covariates

The selection of covariates was based on the findings from preliminary analyses, as well as previously published fundamental cross-sectional studies related to osteoporosis [7, 22–24]. Essential covariates collected in this study included information on demographics (age, gender, education, marital status, and the ratio of family income to poverty (PIR)), behavioral factors (including physical activity, smoking), medical conditions (including diabetes, prednisone or cortisone intake), menstrual status, healthy eating status (including vitamin D supplement, milk consumption), and serum markers (including albumin, alkaline phosphatase, total calcium, and phosphorus). Age was divided into three groups based on the pivotal time points for BMD [9, 25], including ≤ 30, 30–50, and ≥ 50. Race/ethnicity included five groups, including Mexican American, other Hispanic, non-Hispanic white, non-Hispanic black, and others. The education level was classified into three categories as previously described [26, 27]: less than high school, high school or equivalent, and some college or above. Marital status was sorted into three groups, namely married/living with partner, never married and others. BMI was divided into three groups based on the criterion from World Health Organization [28], including normal or low body weight (< 25 kg/m^2^), overweight (25–30 kg/m^2^), and obese (> 30 kg/m^2^). Based on the answer to the question of “Past 30 day milk product consumption”, milk consumption was divided into three categories, including never/rarely (never or less than once a week), sometimes (once or more a week but less than once a day), and often (once a day or more) [19]. Total vitamin D supplement intake (including vitamin D2 and vitamin D3) was determined according to the participants’ records of dietary supplement use in the past 30 days [19]. The confirmation of prednisone or cortisone intake was based on the answer to the question of “Have you ever taken any prednisone or cortisone pills nearly every day for a month or longer?”, and those who answered “yes” were recorded as positive [19]. The weekly metabolic equivalent task (MET)-minute aggregated scores were utilized to evaluate the energy expenditure of physical activity (PA). 8.0 MET scores were assigned for one minute of vigorous work-related activity and vigorous leisure-time physical activity, while 4.0 MET scores were assigned for one minute of moderate work-related activity, walking or bicycling for transportation, and moderate leisure-time (recreational) physical activity. The sum of the above-mentioned five types of PA was the weekly total MET [19]. Participants were divided into four groups based on the PA levels as previously described [14, 29], including inactive (with no moderate- or vigorous-intensity PA), insufficiently active (with total MET being 0 to 600 METs-min), moderate active (total METs being 600 to 3000), and highly active (total METs more than 3000). Smoking status was evaluated by the answer to the question of “Have you smoked at least 100 cigarettes in your entire life?”, and those who answered “yes” were defined as smokers, otherwise defined as nonsmokers or missing group. As previously described and according to the diabetes diagnostic criteria from American Diabetes Association (ADA), participants can be identified as diabetes by the following criteria, including self-reported diagnosis, use of insulin or oral hypoglycemic medication, FBG ≥ 126 mg/dL or HbA1c level ≥ 6.5% [30, 31]. The definition of menopausal status was based on the self-reported reproductive health questionnaire (https://wwwn.cdc.gov/Nchs/Nhanes/2013-2014/RHQ_H.htm#RHQ031). Females were defined as postmenopausal when they meet the following two criteria [32]: (1) firstly answered “no” to the question of “Have you had at least one menstrual period in the past 12 months?”, and (2) then answered “hysterectomy” or “menopause/change of life” to the question of “What is the reason that you have not had a period in the past 12 months?”. The serum markers, including albumin, alkaline phosphatase, total calcium, and phosphorus, were collected from the standard biochemistry profile (https://wwwn.cdc.gov/Nchs/Nhanes/2013-2014/BIOPRO_H.htm).

### Network Pharmacological analysis

Since bone mass or bone homeostasis is primarily fine-tuned by the processes of osteogenesis (bone formation) and osteoclastogenesis (bone resorption) [33, 34], the potential mechanisms of riboflavin regulating bone health were thus explored by network pharmacological analysis. Firstly, the two/three-dimensional and SMILES of riboflavin was obtained from the PubChem database (https://pubchem.ncbi.nlm.nih.gov), and the SMILES was imported into the SuperPred (https://prediction.charite.de/index.php) for target prediction. The predicted targets were further corrected by Uniprot (https://www.uniprot.org/) database to obtain the gene information of drug targets. Similarly, the protein targets related to the two critical processes of bone homeostasis are retrieved from the GeneCards database (https://www.genecards.org/) and Online Mendelian Inheritance in Man (OMIM) database using the key words of “osteogenesis”, “bone formation”, “osteoclastogenesis”, and “bone resorption”. Subsequently, the intersection targets between riboflavin, osteoclastic related target genes, and osteogenic related target genes were obtained using Venny 2.1 (https://bioinfogp.cnb.csic.es/tools/venny/). Then, the protein-protein interactions (PPIs) of the overlapping genes were analyzed in the STRING database (ttps://string-db.org/), and the PPI network was constructed by Cytoscape (Version 3.8.2) software. Furthermore, the selection of core targets was achieved by analyzing the network degree and various parameters with the assistance of network analyzer. The selection of the core targets was based on the inclusion criteria being set at double median degree of freedom, median betweenness centrality and median closeness centrality [35]. Finally, the obtained core targets were imported into the Database for Annotation, Visualization and Integrated Discovery (DAVID, http://david.abcc.ncifcrf.gov/) for Kyoto Encyclopedia of Genes and Genomes (KEGG) and Gene Ontology (GO) analysis. GO terms and KEGG pathways with a P-value of less than 0.05 were considered significant.

### Statistical analysis

Considering the characteristics of complex, multistage sampling design of the NHANES, appropriate sample weights, strata as well as cluster variables were employed. Continuous variables were described as weighted median (Q1-Q3, interquartile) due to their skewed distribution and categorical variables were reported as numbers (weighted proportion, %). Mann-Whitney U test and Chi-square test were utilized to compare the differences between intergroups.

Multivariable logistic regression models were applied to investigate the associations between dietary vitamin B2 intake and osteoporosis and hip fracture, while linear regression models were employed to assess the association between dietary vitamin B2 intake and BMD. One unadjusted model (model 1) combined with two adjusted models (model 2 and model 3) were established. No covariate was adjusted in model 1. Model 2 was adjusted for demographic covariates, including age, race, education, marital status, and PIR. Additional variables, including BMI, prednisone or cortisone intake, milk consumption, vitamin D supplement, diabetes, smoking, menopause, total MET, serum albumin, serum ALP, serum total calcium, serum phosphorus, and energy intake were adjusted in model 3 on the basis of model 2. Additionally, subgroup analyses, interaction tests and restricted cubic spline (RCS) regression analyses were further conducted to explore the robustness and nonlinearity of the association between dietary vitamin B2 intake and osteoporosis.

All statistical analyses were conducted using R software (version 4.3.1), and a P value less than 0.05 was deemed as statistically significant for all two-tailed tests.

## Results

### Sample characteristics

A total of 4, 241 female participants from four cycles of NHANES were included in this study for the association analysis of dietary vitamin B2 intake, osteoporosis, BMD and hip fracture. The baseline characteristics of the included female participants are presented in Table 1, and the differences in dietary vitamin B2 intake, femur BMD in different regions, and hip fracture frequency between OP and non-OP groups were reported in Table. S2 and Fig. S1. Overall, age, race, education, marital status, BMI, total MET, smoking, milk consumption, prednisone or cortisone intake, diabetes, menopausal status, serum markers (including albumin, ALP, total calcium, and phosphorus), and dietary intake of energy and nutrients (including calcium, magnesium, vitamin D and zinc) were statistically significant between OP and non-OP groups (Table 1). Specifically, compared with non-OP participants, OP participants were more likely to be older, be Non-Hispanic White, be postmenopausal, suffer diabetes, with lower levels in education, BMI and total MET, with history of prednisone or cortisone intake, and showing less intake of energy, calcium, magnesium, vitamin D and zinc (all P value < 0.05, Table 1). Additionally, as opposed to non-OP participants, OP individuals showed remarkably lower levels of BMD in different femur regions (all P value < 0.05, Table. S2), as well as reduced levels of dietary vitamin B2 intake (P value < 0.05, Fig.S1), while presented higher percentages in hip fracture (P value < 0.05, Table. S2).

**Table 1:** Baseline characteristics of survey participants according to osteoporosis diagnosis

Characteristics	Overall (*n* = 4241)	Non-osteoporosis(*n* = 2966)	Osteoporosis(*n* = 1275)	*P*-value
**Age**	50.00 (38–62)	45.00 (35–57)	60 (50–70)	< 0.001
**Age categorical**				< 0.001
<=30	618 (14.57%)	539 (18.17%)	79 (6.20%)	
30–50	1570 (37.02%)	1318 (44.44%)	252 (19.76%)	
>=50	2053 (48.41%)	1109 (37.39%)	944 (74.04%)	
**Race/ethnicity**				< 0.001
Mexican American	717 (16.91%)	518 (17.46%)	199 (15.61%)	
Other Hispanic	467 (11.01%)	351 (11.83%)	116 (9.10%)	
Non-Hispanic White	1968 (46.40%)	1294 (43.63%)	674 (52.86%)	
Non-Hispanic Black	762 (17.97%)	611 (20.60%)	151 (11.84%)	
Other Race	327 (7.71%)	192 (6.47%)	135 (10.59%)	
**Education**				0.025
Less than high school	970 (22.87%)	655 (22.08%)	315 (24.71%)	
High school or equivalent	932 (21.98%)	635 (21.41%)	297 (23.29%)	
Some college or above	2339 (55.15%)	1676 (56.51%)	663 (52.00%)	
**Marital status**				< 0.001
Married/living with partner	2431 (57.32%)	1752 (59.07%)	679 (53.25%)	
Never married	608 (14.34%)	497 (16.76%)	111 (8.71%)	
Others	1202 (28.34%)	717 (24.17%)	485 (38.04%)	
**PIR**	2.16 (1.11–4.18)	2.18 (1.10–4.24)	2.14 (1.14–4.12)	0.836
**BMI**	27.36 (23.60-31.96)	28.70 (24.69–33.40)	24.90 (21.77–28.30)	< 0.001
**BMI categorical**				< 0.001
<=25	1435 (33.84%)	787 (26.53%)	648 (50.82%)	
25–30	1339 (31.57%)	928 (31.29%)	411 (32.24%)	
>=30	1467 (34.59%)	1251 (42.18%)	216 (16.94%)	
**Total MET**	800 (0-2760)	920.00 (0-3070)	560 (0-1920)	< 0.001
**Total MET categorical**				< 0.001
Inactive	1217 (28.70%)	788 (26.57%)	429 (33.65%)	
Insufficiently active	610 (14.38%)	411 (13.86%)	199 (15.61%)	
Moderate active	537 (12.66%)	361 (12.17%)	176 (13.80%)	
Highly active	1877 (44.26%)	1406 (47.40%)	471 (36.94%)	
**Vitamin D supplement**,** mg**	0.02 (0.02–0.02)	0.02 (0.02–0.02)	0.02 (0.02–0.02)	0.897
**Milk consumption**				< 0.001
Never/rarely (never or less than once a week)	1459 (34.40%)	970 (32.70%)	489 (38.35%)	
Sometimes (once or more a week but less than once a day)	1219 (28.74%)	899 (30.31%)	320 (25.10%)	
Often (once a day or more)	1563 (36.85%)	1097 (36.99%)	466 (36.55%)	
**Prednisone or cortisone intake**				< 0.001
Yes	270 (6.37%)	161 (5.43%)	109 (8.55%)	
No	3971 (93.63%)	2805 (94.57%)	1166 (91.45%)	
**Diabetes**				< 0.001
Yes	448 (10.56%)	277 (9.34%)	171 (13.41%)	
No	3793 (89.44%)	2689 (90.66%)	1104 (86.59%)	
**Smoking**				0.427
No	767 (18.09%)	525 (17.70%)	242 (18.98%)	
Yes	785 (18.51%)	542 (18.27%)	243 (19.06%)	
Missing	2689 (63.40%)	1899 (64.03%)	790 (61.96%)	
**Menopause**				< 0.001
No	2077 (48.97%)	1770 (59.68%)	307 (24.08%)	
Yes	2164 (51.03%)	1196 (40.32%)	968 (75.92%)	
**Albumin**,** g/L**	42.00 (40.00–44.00)	42.00 (40.00–44.00)	42.00 (40.00–44.00)	0.001
**ALP**,** U/L**	66.00 (53.00–81.00)	63.00 (51.00–79.00)	71.00 (57.00–86.00)	< 0.001
**Serum total calcium**,** mmol/L**	2.350 (2.300–2.400)	2.350 (2.300-2.425)	2.350 (2.300–2.400)	< 0.001
**Phosphorus**,** mmol/L**	1.227 (1.098–1.324)	1.259 (1.130–1.356)	1.227 (1.098–1.324)	< 0.001
**Dietary intake**				
**Energy**,** kcal**	1940.00 (1433.00-2619.00)	2004.50 (1492.00-2703.00)	1688.00 (1263.50–2252.00)	< 0.001
**Riboflavin**,** mg**	1.86 (1.30–2.58)	1.92 (1.32–2.65)	1.69 (1.20–2.30)	< 0.001
**Calcium**,** mg**	817.00 (529.00-1184.00)	837.00 (541.00-1213.00)	737.00 (479.00-1050.00)	< 0.001
**Magnesium**,** mg**	270.00 (197.00-367.00)	278.00 (202.00-375.00)	245.00 (178.00-333.00)	< 0.001
**Vitamin D**,** mg**	3.20 (1.30–6.10)	3.20 (1.30–6.20)	3.00 (1.20–5.40)	0.001
**Zinc**,** mg**	9.81 (6.78–14.18)	10.21 (7.02–14.70)	8.58 (5.94–12.25)	< 0.001

Abbreviations: RCS, restricted cubic spline; BMI, body mass index; MET, metabolic equivalent task; PIR, ratio of family income to poverty; ALP, alkaline phosphatase

### Association between dietary vitamin B2 intake and osteoporosis, BMD, and hip fracture

To determine the association between dietary vitamin B2 intake and osteoporosis and hip fracture, multiple logistic regression models were utilized and the results were presented in Tables 2 and 3, respectively. No significant association was observed between dietary vitamin B2 intake and hip fracture in unadjusted, partially- or fully-adjusted models (Table 3). In contrast, in model 3, the intake of vitamin B2 was found to be significantly correlated with decreased risks for osteoporosis regardless of whether the intake of vitamin B2 was analyzed as continuous variable or categorical variable (Table 2). In model 3, the highest quantile of vitamin B2 intake (OR = 0.613; 95%CI: 0.454–0.829) was positively associated with decreased risk of osteoporosis compared to quantile 1 (P for trend = 0.002).

**Table 2:** Association between dietary total vitamin B2 intake and osteoporosis

Characteristics	Model 1 OR (95%CI) *P*-value	Model 2 OR (95%CI) *P*-value	Model 3 OR (95%CI) *P*-value
**Vitamin B2**	0.942 (0.879, 1.009) 0.08914	0.926 (0.856, 1.002) 0.05585	0.893 (0.806, 0.990) **0.03209**
**Vitamin B2 quartile**			
Q1	Reference	Reference	Reference
Q2	0.971 (0.821, 1.150) 0.73658	0.829 (0.688, 0.998) **0.04795**	0.826 (0.669, 1.020) 0.07557
Q3	0.922 (0.773, 1.100) 0.36681	0.796 (0.655, 0.968) **0.02246**	0.800 (0.631, 1.013) 0.06405
Q4	0.765 (0.621, 0.941) **0.01122**	0.712 (0.566, 0.897) **0.00390**	0.613 (0.454, 0.829) **0.00148**
P for trend	**0.016**	**0.002**	**0.002**

Abbreviations: BMI, body mass index; MET, metabolic equivalent task; PIR, ratio of family income to poverty; ALP, Alkaline phosphatase, OR, odds ratio; CI, confidence interval. Model 1, no covariates were adjusted. Model 2, demographic covariates were adjusted, including age, race, education, marital status, and PIR. Model 3, based on Model 2, was additionally adjusted for covariates of BMI, prednisone or cortisone intake, milk consumption, Vitamin D supplement, diabetes, smoking, menopause, total MET, serum albumin, serum ALP, serum total calcium, serum phosphorus, and energy intake

**Table 3:** Association between dietary total vitamin B2 intake and femoral BMD

Characteristics	Model 1 β (95%CI) *P*-value	Model 2 β (95%CI) *P*-value	Model 3 β (95%CI) *P*-value
Total femur BMD			
Vitamin B2	0.004 (-0.000, 0.009) 0.062	0.006 (0.002, 0.010) **0.004**	0.008 (0.003, 0.012) **0.001**
Vitamin B2 quartile			
Q1	Reference	Reference	Reference
Q2	-0.003 (-0.014, 0.009) 0.625	0.009 (-0.001, 0.019) 0.080	0.006 (-0.004, 0.015) 0.242
Q3	0.002 (-0.009, 0.014) 0.688	0.014 (0.003, 0.025) **0.011**	0.010 (-0.000, 0.021) 0.061
Q4	0.014 (0.001, 0.028) **0.037**	0.019 (0.007, 0.031) **0.002**	0.020 (0.007, 0.033) **0.002**
P for trend	0.051	**0.001**	**0.017**
**Femoral neck BMD**			
Vitamin B2	0.002 (-0.003, 0.006) 0.430	0.004 (0.000, 0.008) **0.046**	0.005 (0.001, 0.009) **0.017**
Vitamin B2 quartile			
Q1	Reference	Reference	Reference
Q2	-0.009 (-0.020, 0.003) 0.135	0.008 (-0.002, 0.017) 0.119	0.005 (-0.004, 0.014) 0.289
Q3	-0.003 (-0.015, 0.009) 0.658	0.013 (0.003, 0.023) **0.010**	0.010 (-0.000, 0.020) 0.050
Q4	0.007 (-0.007, 0.021) 0.317	0.013 (0.002, 0.025) **0.025**	0.015 (0.002, 0.027) **0.022**
P for trend	0.394	**0.007**	**0.041**
**Trochanter BMD**			
Vitamin B2	0.005 (0.001, 0.009) **0.007**	0.006 (0.002, 0.009) **0.001**	0.007 (0.003, 0.011) **0.001**
Vitamin B2 quartile			
Q1	Reference	Reference	Reference
Q2	0.002 (-0.007, 0.011) 0.675	0.010 (0.001, 0.019) **0.025**	0.007 (-0.001, 0.015) **0.090**
Q3	0.004 (-0.006, 0.013) 0.457	0.011 (0.001, 0.020) **0.023**	0.007 (-0.002, 0.017) 0.116
Q4	0.018 (0.007, 0.029) **0.001**	0.019 (0.009, 0.030) **0.000**	0.020 (0.009, 0.031) **0.000**
P for trend	0.126	**0.009**	**0.008**
**Intertrochanter BMD**			
Vitamin B2	0.004 (-0.001, 0.010) 0.126	0.007 (0.002, 0.011) **0.009**	0.009 (0.003, 0.014) **0.001**
Vitamin B2 quartile			
Q1	Reference	Reference	Reference
Q2	-0.004 (-0.017, 0.010) 0.567	0.009 (-0.003, 0.021) 0.149	0.005 (-0.006, 0.017) 0.372
Q3	0.002 (-0.012, 0.016) 0.807	0.015 (0.002, 0.028) **0.025**	0.011 (-0.002, 0.023) 0.103
Q4	0.013 (-0.003, 0.029) 0.101	0.020 (0.005, 0.035) **0.007**	0.022 (0.006, 0.037) **0.007**
P for trend	0.126	**0.003**	**0.038**

Abbreviations: BMD, bone mineral density; BMI, body mass index; MET, metabolic equivalent task; PIR, ratio of family income to poverty; ALP, Alkaline phosphatase, OR, odds ratio; CI, confidence interval. Model 1, no covariates were adjusted. Model 2, demographic covariates were adjusted, including age, race, education, marital status, and PIR. Model 3, based on Model 2, was additionally adjusted for covariates of BMI, prednisone or cortisone intake, milk consumption, Vitamin D supplement, diabetes, smoking, menopause, total MET, serum albumin, serum ALP, serum total calcium, serum phosphorus, and energy intake

**Table 4:** Association between dietary total vitamin B2 intake and hip fracture

Characteristics	Model 1 OR (95%CI) *P*-value	Model 2 OR (95%CI) *P*-value	Model 3 OR (95%CI) *P*-value
**Vitamin B2**	1.001 (0.770, 1.301) 0.993	0.994 (0.759, 1.301) 0.963	1.203 (0.915, 1.582) 0.184
**Vitamin B2 quartile**			
Q1	Reference	Reference	Reference
Q2	0.832 (0.424, 1.634) 0.593	0.811 (0.408, 1.610) 0.549	1.134 (0.549, 2.341) 0.733
Q3	0.960 (0.489, 1.884) 0.905	0.967 (0.483, 1.934) 0.923	1.541 (0.708, 3.350) 0.275
Q4	0.991 (0.461, 2.129) 0.981	0.977 (0.443, 2.151) 0.953	1.903 (0.738, 4.906) 0.182
P for trend	0.988	0.991	0.143

Abbreviations: BMI, body mass index; MET, metabolic equivalent task; PIR, ratio of family income to poverty; ALP, Alkaline phosphatase, OR, odds ratio; CI, confidence interval. Model 1, no covariates were adjusted. Model 2, demographic covariates were adjusted, including age, race, education, marital status, and PIR. Model 3, based on Model 2, was additionally adjusted for covariates of BMI, prednisone or cortisone intake, milk consumption, Vitamin D supplement, diabetes, smoking, menopause, total MET, serum albumin, serum ALP, serum total calcium, serum phosphorus, and energy intake

General linear regression models were used to explore the association of vitamin B2 intake and BMD, with the results being presented in Table 4. Similarly, the intake of vitamin B2 was found to be positively associated with increased levels of femur BMD in model 2 and model 3, regardless of the variable type of vitamin B2 intake. In model 3, when vitamin B2 intake was analyzed as continuous variable, 1 mg increment of vitamin B2 intake was associated with conspicuous elevation of BMD in all femur regions, with the β being 0.008 (95%CI: 0.003–0.012), 0.005 (95%CI: 0.001–0.009), 0.007 (95%CI: 0.003–0.011) and 0.009 (95%CI: 0.003–0.014) for total femur BMD, femoral neck BMD, trochanter BMD, and intertrochanter BMD, respectively (all P value < 0.05, Table 4). Consistently, when vitamin B2 intake was analyzed as categorical variable, the quantile 4 group was positively associated with increased BMD in all femur regions when compared with the quantile 1 group, with the β being 0.020 (95%CI: 0.007–0.033), 0.015 (95%CI: 0.002–0.027), 0.020 (95%CI: 0.009–0.031) and 0.022 (95%CI: 0.006–0.037) for total femur BMD, femoral neck BMD, trochanter BMD, and intertrochanter BMD, respectively (all P value < 0.05, all P for trend < 0.05, Table 4).

### Identification of potential nonlinear relationships between dietary vitamin B2 intake and osteoporosis, BMD, and hip fracture

**Fig. 2:**
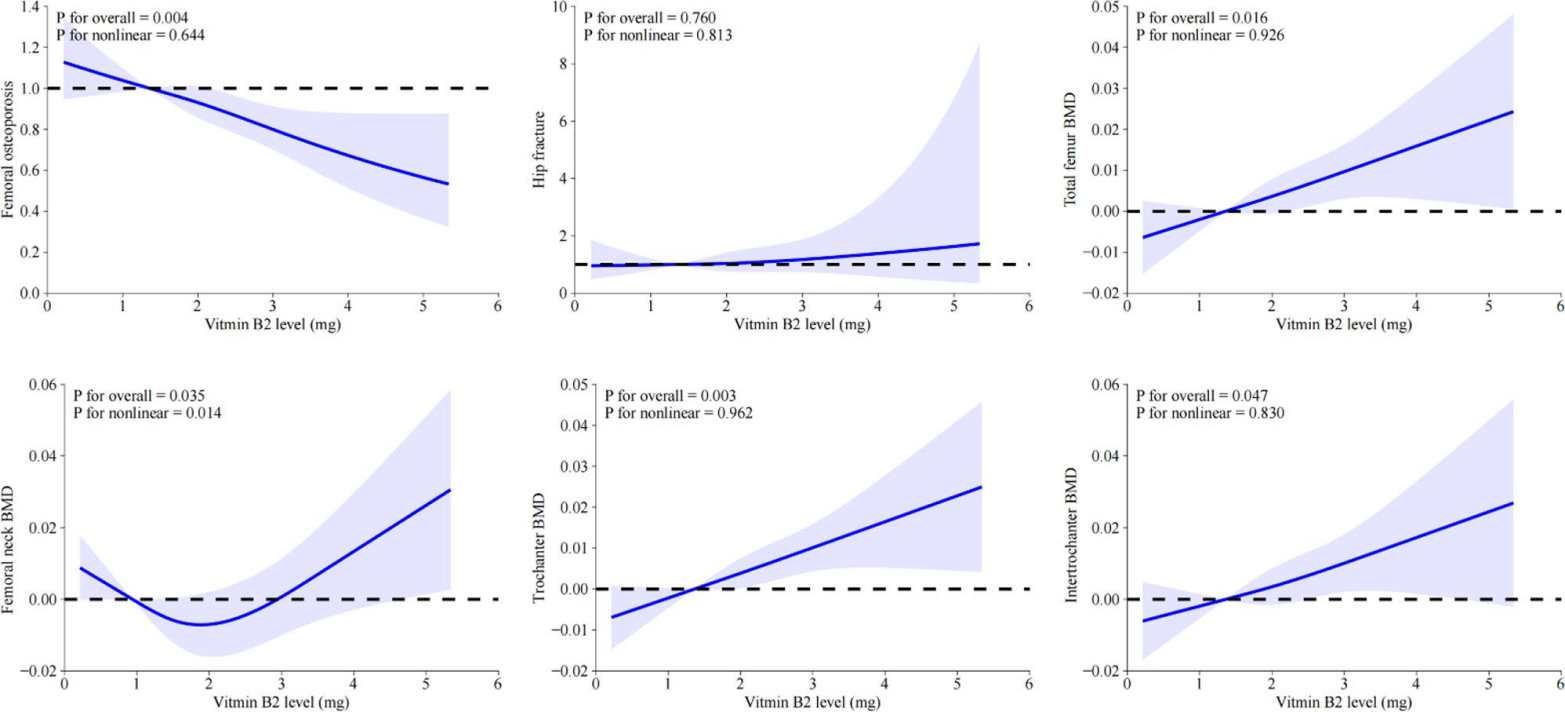
RCS analysis exploring the association between dietary intake of Vitamin B2 level and osteoporosis, hip fracture and femoral BMD in all participants. Variables of age, race, education, marital status, PIR, BMI, prednisone or cortisone intake, milk consumption, Vitamin D supplement, diabetes, smoking, menopause, total MET, serum albumin, serum ALP, serum total calcium, and serum phosphorus were adjusted during RCS analyses. Abbreviations: RCS, restricted cubic spline; BMI, body mass index; MET, metabolic equivalent task; PIR, ratio of family income to poverty; ALP, alkaline phosphatase

The above-mentioned results indicated that the dietary vitamin B2 intake, whether being continuous variable or categorical variable, was significantly associated with reduced risks for osteoporosis and increased femur BMD, while not significantly related to the risk of hip fracture. Therefore, multivariate restricted cubic spline (RCS) analysis was used to determine the potential nonlinear relationships between dietary vitamin B2 intake and osteoporosis, BMD, and hip fracture. As depicted in Fig. 2, vitamin B2 intake was found to be negatively and linearly associated with risks for femoral OP (P for overall = 0.004, P for nonlinear = 0.644), and positively and linearly correlated with total femur BMD (P for overall = 0.016, P for nonlinear = 0.926), trochanter BMD (P for overall = 0.003, P for nonlinear = 0.962) and intertrochanter BMD (P for overall = 0.047, P for nonlinear = 0.830), while positively and nonlinearly correlated with femoral neck BMD (P for overall = 0.035, P for nonlinear = 0.014). However, no significant relationship was found between vitamin B2 intake and hip fracture (P for overall = 0.760, P for nonlinear = 0.813).

### Association between dietary vitamin B2 intake, osteoporosis and femur BMD levels within different subgroups

**Fig. 3:**
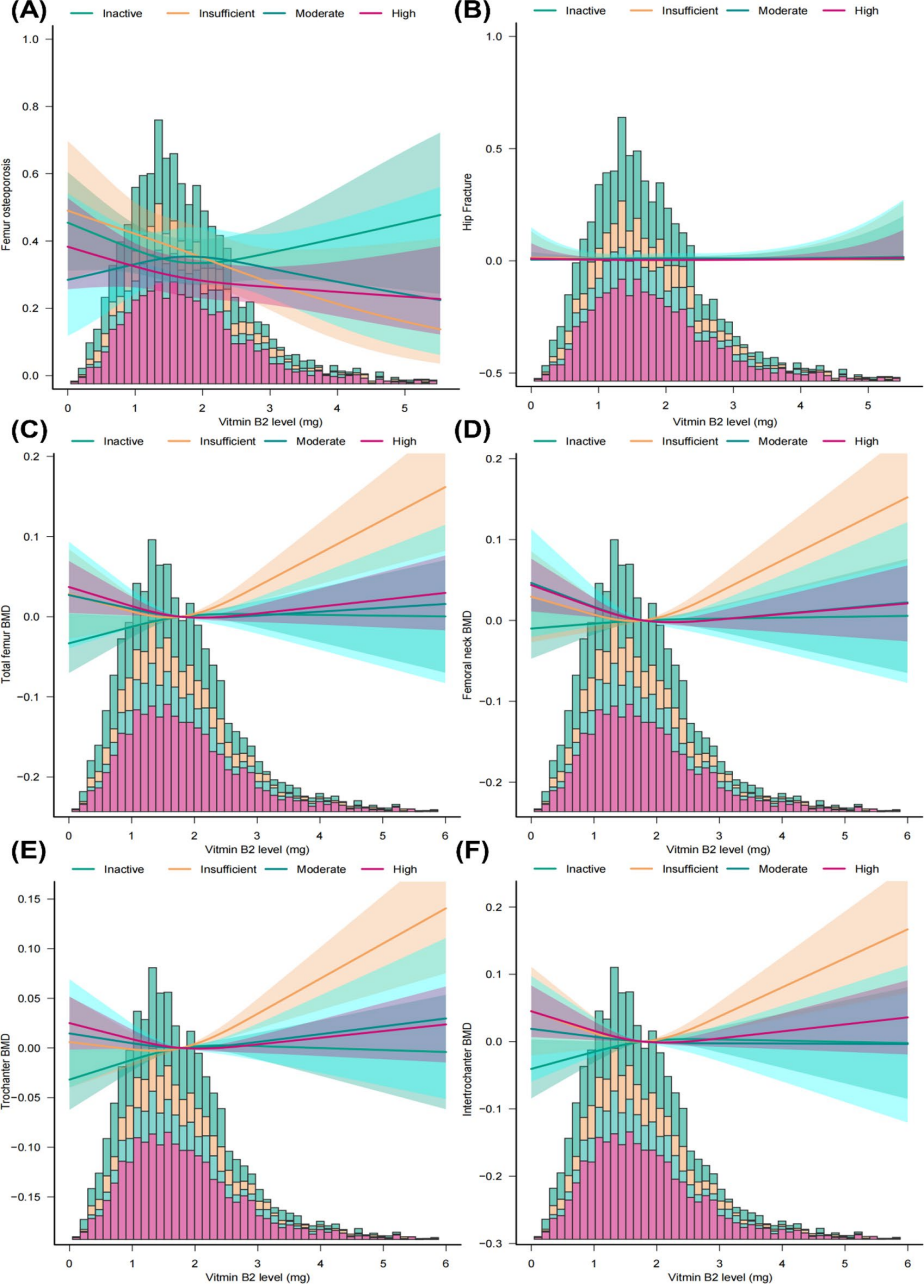
RCS analysis exploring the association between dietary intake of Vitamin B2 level and osteoporosis, hip fracture and femoral BMD in different total MET subgroups. (**A**) Femur osteoporosis, (**B**) Hip fracture, (**C**) Total femur BMD, (**D**) Femoral neck BMD, (**E**) Trochanter BMD, and (**F**) Intertrochanter BMD. Variables of age, race, education, marital status, PIR, BMI, prednisone or cortisone intake, milk consumption, Vitamin D supplement, diabetes, smoking, menopause, serum albumin, serum ALP, serum total calcium, and serum phosphorus were adjusted during RCS analyses. Abbreviations: RCS, restricted cubic spline; BMI, body mass index; MET, metabolic equivalent task; PIR, ratio of family income to poverty; ALP, alkaline phosphatase

In order to further confirm the robustness of the above analytic results, subgroup analyses and interaction tests were subsequently performed. As presented in Table 5, except for the variable of total MET, the relationship between vitamin B2 intake and osteoporosis remained consistent in different subgroups stratified by age, BMI, race, education level, marital status, menopausal status, milk consumption, diabetes and smoking. Moreover, interaction tests indicated that the associations between vitamin B2 intake and osteoporosis was modified by the variable of total MET, with the risk for OP reduced more pronounced in the subgroup of insufficiently active individuals as the increment of vitamin B2 intake (P interaction = 0.0364, Table 5). Additionally, the RCS analyses based on different total MET groups also verified the findings from subgroup analyses, with a more pronounced relationship between vitamin B2 intake and osteoporosis and femur BMD being observed in insufficiently active participants (Fig. 3). Similarly, the association between vitamin B2 intake and BMD levels in different femur regions was also verified by subgroup and interaction analyses. As presented in Table S3, the association remained stable in different subgroups except for the stratifying variable of education and milk consumption, with the aforementioned association being more pronounced in those with higher education levels (P interaction = 0.0413 for total femur BMD, and P interaction = 0.0108 for intertrochanter BMD) and with more milk consumption (P interaction = 0.0405 for trochanter BMD).

**Table 5:** Association between dietary total vitamin B2 intake and osteoporosis in different subgroups

Characteristics	OR (95%CI) *P*-value	*P* interaction
**Age**		0.7109
<=30	0.921 (0.716, 1.185) 0.522	
30–50	0.801 (0.679, 0.944) 0.008	
>=50	0.905 (0.810, 1.012) 0.079	
**BMI**		0.5406
<=25	0.898 (0.797, 1.013) 0.079	
25–30	0.829 (0.708, 0.969) 0.018	
>=30	0.926 (0.752, 1.139) 0.465	
**Total MET**		**0.0364**
Inactive	0.991 (0.842, 1.166) 0.915	
Insufficiently active	0.623 (0.477, 0.814) < 0.001	
Moderate active	0.910 (0.702, 1.178) 0.473	
Highly active	0.892 (0.787, 1.010) 0.072	
**Race**		0.9813
Mexican American	0.910 (0.733, 1.130) 0.393	
Other Hispanic	0.878 (0.648, 1.190) 0.401	
Non-Hispanic White	0.850 (0.755, 0.956) 0.007	
Non-Hispanic Black	0.895 (0.719, 1.115) 0.323	
Other Race	0.912 (0.641, 1.299) 0.611	
**Education**		0.2989
Less than high school	0.981 (0.830, 1.161) 0.824	
High school or equivalent	0.831 (0.693, 0.997) 0.045	
Some college or above	0.847 (0.748, 0.959) 0.008	
**Marital Status**		0.2685
Married/living with partner	0.920 (0.820, 1.032) 0.153	
Never married	0.864 (0.682, 1.094) 0.224	
Others	0.804 (0.685, 0.943) 0.007	
**Menopause**		0.3315
No	0.834 (0.723, 0.962) 0.012	
Yes	0.909 (0.815, 1.014) 0.088	
**Prednisone or cortisone intake**		0.2563
Yes	0.697 (0.483, 1.006) 0.053	
No	0.893 (0.818, 0.975) 0.011	
**Milk consumption**		0.5615
Never/rarely (never or less than once a week)	0.910 (0.780, 1.062) 0.231	
Sometimes (once or more a week but less than once a day)	0.940 (0.786, 1.125) 0.501	
Often (once a day or more)	0.820 (0.718, 0.936) 0.003	
**Diabetes**		0.4123
Yes	0.813 (0.630, 1.049) 0.111	
No	0.887 (0.810, 0.972) 0.010	
**Smoking**		0.3131
No	0.850 (0.692, 1.045) 0.123	
Yes	1.057 (0.906, 1.233) 0.480	
Missing	0.812 (0.720, 0.915) < 0.001	

Abbreviations: BMI, body mass index; MET, metabolic equivalent task; PIR, ratio of family income to poverty; ALP, Alkaline phosphatase, OR, odds ratio; CI, confidence interval. The variables adjusted for subgroup analyses were consistent with Model 3 in Table 2 except the stratifying variable

### ALP mediates the association between dietary vitamin B2 intake and osteoporosis and BMD

**Fig. 4:**
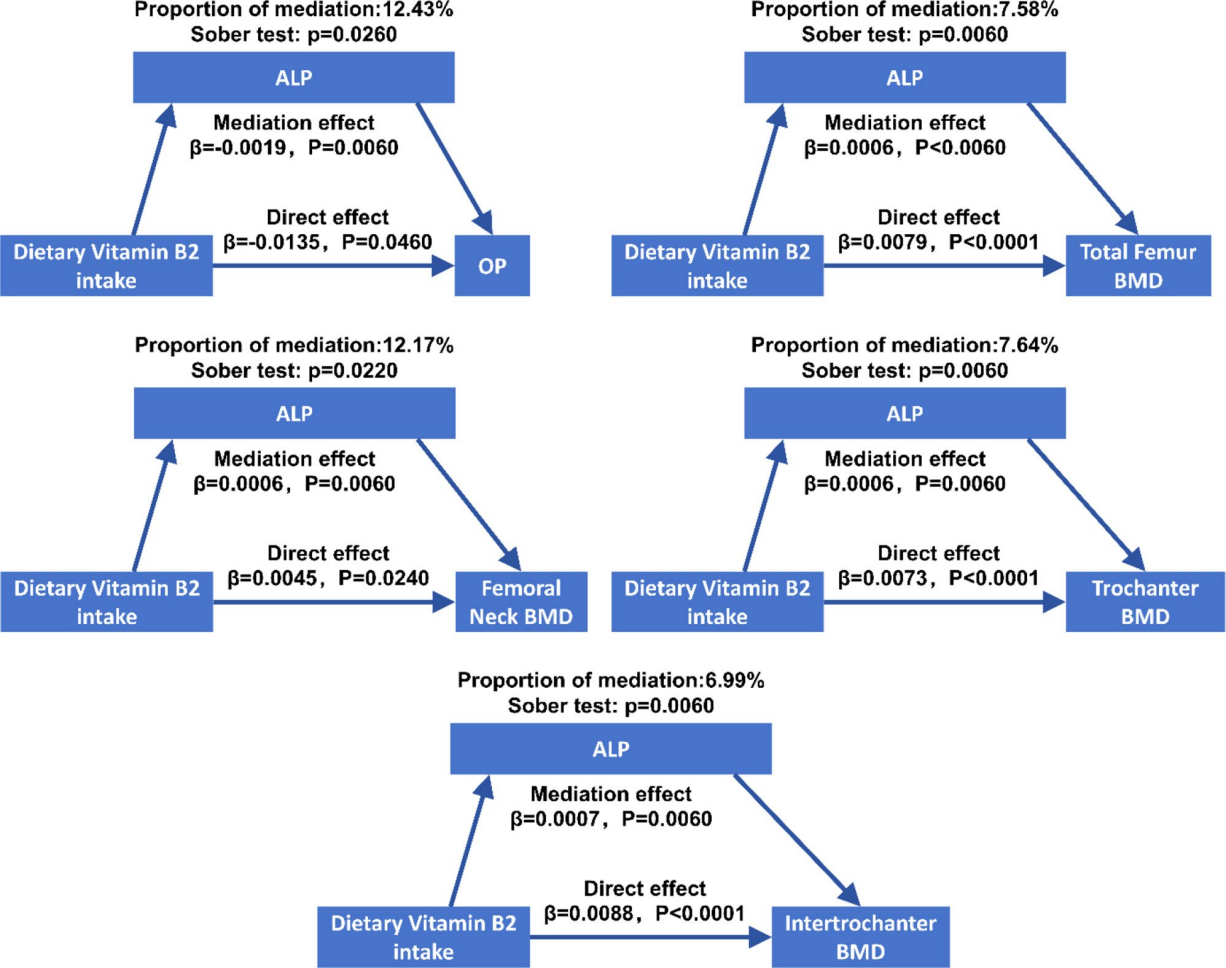
ALP mediated the association between dietary vitamin B2 intake and OP and femur BMD in all regions

The association between dietary vitamin B1 intake and femur OP and BMD were significantly mediated by ALP, with the mediation proportions being 12.43%, 7.58%, 12.17%, 7.64% and 6.99% for femoral OP, total femur BMD, femoral neck BMD, trochanter BMD, and intertrochanter BMD, respectively (Sober test, all *P* < 0.05). The direct effect and mediated effect were − 0.014 (95% CI: -0.027, -0.000) and − 0.002 (95% CI: -0.004, -0.000) for femoral OP, 0.008 (95% CI: 0.004, 0.012) and 0.001 (95% CI: 0.000, 0.001) for total femur BMD, 0.004 (95% CI: 0.000, 0.008) and 0.001 (95% CI: 0.000, 0.001) for femoral neck BMD, 0.007 (95% CI: 0.004, 0.010) and 0.001 (95% CI: 0.000, 0.001) for trochanter BMD, and 0.009 (95% CI: 0.004, 0.014) and 0.001 (95% CI: 0.000, 0.001) for intertrochanter BMD, respectively (all *P* < 0.05, Fig. 4).

### Potential mechanisms and targets of vitamin B2 involved in bone homeostasis

**Fig. 5:**
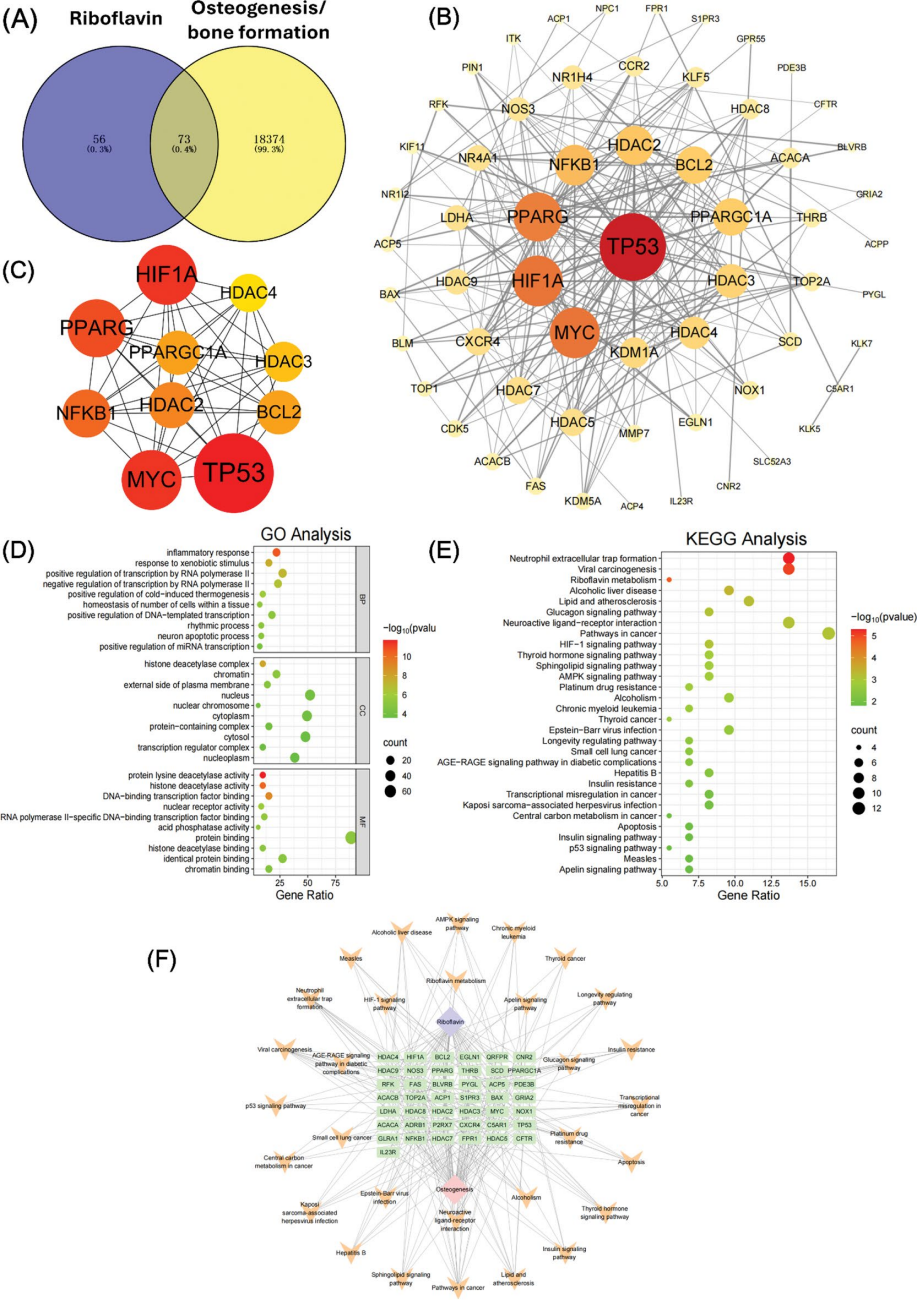
Identification of potential targets and mechanisms of vitamin B2 in regulating osteogenesis through network pharmacological analysis. (**A**) Venn graph showing the intersection targets of vitamin B2 and genes related to osteogenesis/bone formation. (**B**) The protein-protein interaction (PPI) network of overlapping targets. (**C**) The PPI network of the core intersection targets. (**D**) GO enrichment analysis based on core intersection genes. (**E**) KEGG enrichment analysis based on core intersection genes. (**F**) The plot of drug-disease-target genes network established by Cytoscape software

**Fig. 6:**
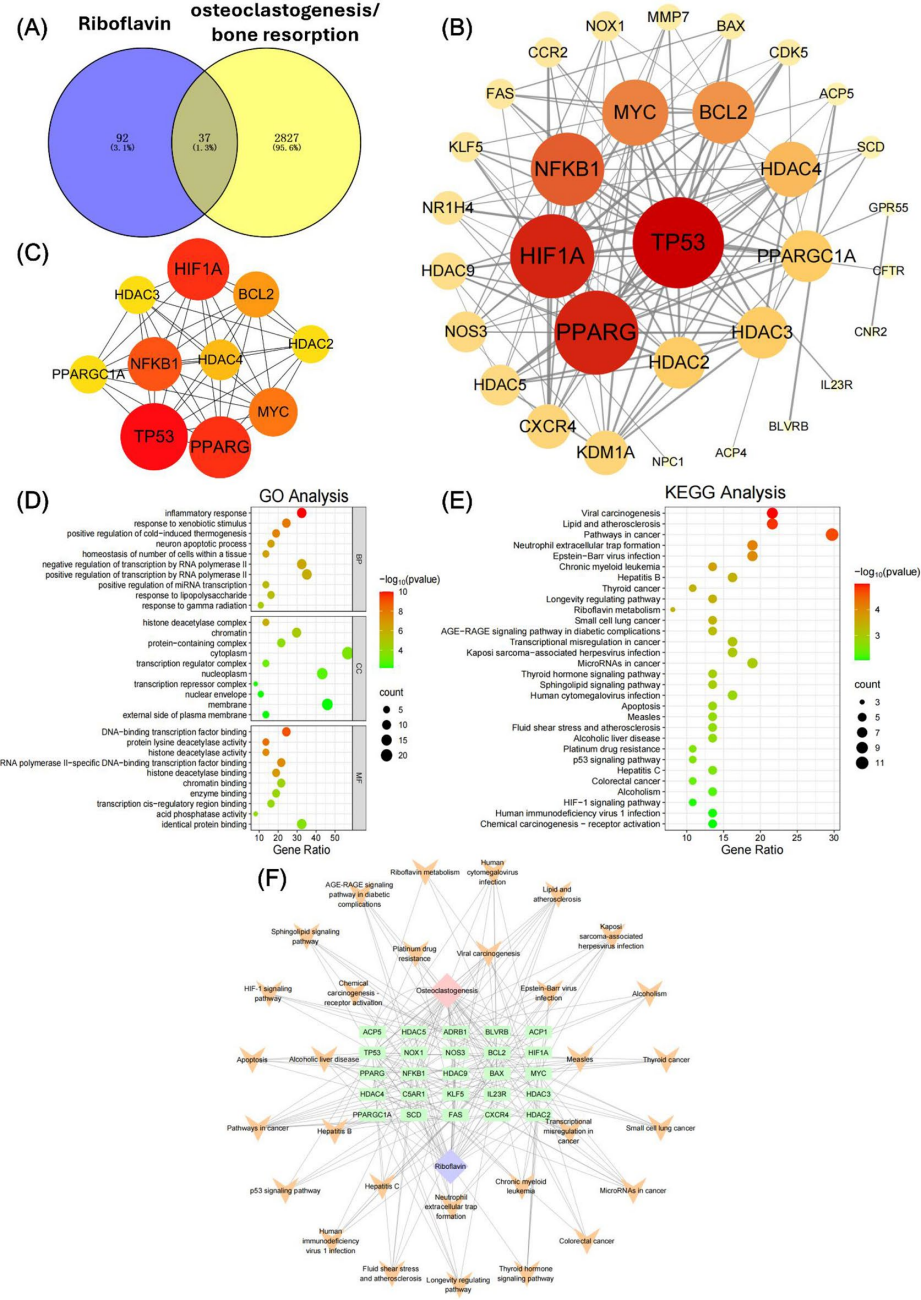
Determination of potential targets and mechanisms of vitamin B2 in regulating osteoclastogenesis through network pharmacological analysis. (**A**) Venn graph showing the intersection targets of vitamin B2 and genes related to osteoclastogenesis/bone resorption. (**B**) The protein-protein interaction (PPI) network of overlapping targets. (**C**) The PPI network of the core intersection targets. (**D**) GO enrichment analysis based on core intersection genes. (**E**) KEGG enrichment analysis based on core intersection genes. (**F**) The plot of drug-disease-target genes network established by Cytoscape software

A total of 129 genes, 18,447 genes and 2864 genes were identified to associate with vitamin B2, osteogenesis/bone formation, and osteoclastogenesis/bone resorption (Figs. 5A and 6A), respectively. 73 intersection targets were determined between vitamin B2 and osteogenesis/bone formation, and 37 overlapping targets were obtained between vitamin B2 and osteoclastogenesis/bone resorption. In addition, a total of 37 overlapping targets were confirmed by Venn analysis between riboflavin, osteogenic, and osteoclastic processes (Fig. S2). The STRING database was then utilized to get the PPI information of the selected 73 and 37 overlapping targets, and then the PPI network (Figs. 5B and 6B), as well as the top 10 hub target genes (Figs. 5C and 6C), were established and evaluated by Cytoscape software. According to the defined criteria for core targets selection, the top 10 core genes for osteogenic/osteoclastic process regulated by riboflavin were HIF1A, HDAC4, HDAC3, HDAC2, BCL2, TP53, MYC, NFKB1, PPARG and PPARGC1A (Figs. 5C and 6C).

The DAVID database was used to perform GO enrichment and KEGG pathway analyses based on the selected core target genes, and the results were presented in Figs. 5 and 6. According to the results, the top 10 entries for GO terms and KEGG pathways were visualized. Specifically, regarding the core targets for osteogenesis regulated by riboflavin, the biological process (BP) mainly focused on inflammatory response, response to xenobiotic stimulus, positive regulation of transcription by RNA polymerase II, negative regulation of transcription by RNA polymerase II, etc., and cell composition (CC) mainly included histone deacetylase complex, external side of plasma membrane, transcription regulator complex, etc., while molecular function (MF) primarily involved in protein lysine deacetylase activity, histone deacetylase activity, DNA-binding transcription factor binding (Fig. 5D), and so on. Additionally, the enriched KEGG pathways based on the aforementioned core target genes mainly included neutrophil extracellular trap formation, HIF-1 signaling pathway, longevity regulating pathway, p53 signaling pathway (Fig. 5E), and so on. The network diagram between riboflavin, osteogenesis and significantly enriched KEGG pathways was further plotted and presented in Fig. 5F.

Similarly, regarding the core targets for osteoclastogenesis regulated by riboflavin, the related terms for BP, CC and MF were inflammatory response, response to xenobiotic stimulus, response to lipopolysaccharide, etc., and histone deacetylase complex, protein-containing complex, transcription regulator complex, etc., and DNA-binding transcription factor binding, protein lysine deacetylase activity, histone deacetylase activity, etc., respectively (Fig. 6D). Moreover, the KEGG pathways associated with these core target genes included neutrophil extracellular trap formation, longevity regulating pathway, riboflavin metabolism, AGE-RAGE signaling pathway in diabetic complications, sphingolipid signaling pathway, p53 signaling pathway, HIF-1 signaling pathway (Fig. 6E), and so on. The relationship between riboflavin, osteoclastogenesis and significantly enriched KEGG pathways, as revealed by the drug-target-pathway network, was shown in Fig. 6F.

## Discussion

In this study, the relationship between dietary riboflavin (vitamin B2) intake and osteoporosis was explored in U.S. female adults. Our results revealed that increased vitamin B2 intake was beneficially associated with reduced risk for osteoporosis and elevated levels of BMD in different femoral regions. Moreover, vitamin B2 intake was found to be significantly, negatively and linearly correlated with odds of osteoporosis, and positively and linearly associated with BMD in regions of total femur, trochanter and intertrochanter, while positively and nonlinearly associated with femoral BMD. Additionally, subgroup analyses and interaction tests suggested that the relationship between vitamin B2 intake and osteoporosis was more pronounced in insufficiently active individuals. Meanwhile, serum ALP levels was found to mediate the association between vitamin B2 intake and osteoporosis and femur BMD levels. Finally, network pharmacological analysis indicated that riboflavin may regulate bone metabolism through pathways like HIF-1 signaling pathway, p53 signaling pathway, AGE-RAGE signaling pathway, longevity regulating pathway, etc.

Consistent with our findings, several studies have also revealed the beneficial association of vitamin B2 intake with osteoporosis and BMD. Nahid Yazdanpanah et al. found that increased dietary riboflavin intake was associated with higher femoral neck BMD in elderly men and women [36]. In the cohort of postmenopausal women, as opposed to those with the 677-CC genotype, those who had the lowest quartile of riboflavin intake and homozygous for the MTHFR 677 T allele were found to reap 1.8 and 2.6 times higher risk for incident osteoporotic fractures fragility fractures [37], respectively. Also, the findings from Bolaji Lilian Ilesanmi-Oyelere et al. suggested that higher levels of riboflavin intakes were positively associated with spine and femoral neck BMD in postmenopausal women [38]. Moreover, a recent clinical investigations based on Chinese adult population (the TCLSIH cohort study) have consistently revealed that dietary riboflavin intake was negatively correlated with the prevalence of osteoporosis in women instead of in men [39]. In the multivariate-adjusted models, the OR (95% CI) in the highest quartile of vitamin B2 intake was 0.47 (0.22, 0.96) as opposed to the first quartile group [39]. Additionally, in animal models, riboflavin deficiency was also found to induce down-regulated expression of key osteogenic markers in the femur, including Runx2, Osterix, and BMP-2/Smad1/5/9 cascade [12]. Further cellular and molecular experiments demonstrates that riboflavin deficiency leads to osteoblast malfunction and blocks bone matrix mineralization mainly through p38 MAPK/BMP-2/Smad1/5/9 pathway [12]. Collectively, the integration of our results and the findings from previous studies clearly demonstrates the pivotal role of riboflavin intake in maintaining normal BMD levels and reducing the risk of osteoporosis from both clinical and preclinical perspectives.

This study yielded an interesting finding: no significant association was observed between vitamin B2 intake and hip fracture risk. Several factors may explain this phenomenon. First, hip fracture risk is influenced by multiple factors. Aging, hormone levels (e.g., estrogen), muscle mass, and fall risk all play crucial roles [40–42]. In this complex context, the impact of nutritional factors, especially individual vitamins, is relatively minor. Second, since this is a cross-sectional study, it cannot explore the long-term cumulative effects of riboflavin metabolism on bone health. Hip fracture is often the result of long-term changes, and a cross-sectional study only provides a snapshot. Third, as an observational study, it is difficult to fully control for all potential confounding variables, such as overall diet quality, exercise habits, and comorbidities. For instance, individuals with high riboflavin intake may also consume more dairy products, which are rich in calcium and vitamin D. The bone-protecting effect may thus be attributed to these other nutrients rather than vitamin B2. Taken together, the complexity of hip fracture risk factors, the limitations of the cross-sectional study design, and the presence of confounding variables may all contribute to the lack of a significant association between dietary vitamin B2 intake and hip fracture risk. Therefore, future cohort or randomized controlled studies with long-term follow-up are needed to explore and confirm this relationship.

Regarding the relations of vitamin B2 intake with osteoporosis and femoral BMD, the findings from subgroup analyses and interaction tests revealed that the associations were more pronounced in individuals who were insufficiently active, higher-educated, and consuming more milk products. A significant research performed by Kévin Contrepois, et al. revealed that just ten minutes of acute physical activity can trigger thousands of molecular changes related to inflammation, energy metabolism, oxidative stress, and so on [43]. Since insufficient physical activity was reported to induce lipometabolic disturbance and oxidative stress [44], it is speculated that vitamin B2 may improve bone health via inhibiting this process. That’s a possible reason for the more pronounced association of vitamin B2 intake with osteoporosis and BMD. Furthermore, our study also revealed that individuals with higher education were more likely to benefit from vitamin B2 intake, which was consistent with previous findings [45]. Better education may potentially lower the risk of bone density loss via influencing the knowledge of osteoporosis and thus increasing the possibility of taking interventional and preventional strategies for osteoporosis [45, 46]. Additionally, previous studies suggested that regular milk consumption throughout life was found to improve bone mineral content (BMC) and BMD in old age without gender differences [47, 48]. Adequate milk consumption can provide abundant essential proteins or amino acids for bone metabolism [49], which may enhance the effects of vitamin B2 intake on osteoporosis and BMD.

Similar to other B vitamins, riboflavin facilitates energy production by assisting in the metabolism of fats, carbohydrates, and proteins. It is a vital component of the cofactors flavin adenine dinucleotide (FAD) and flavin mononucleotide (FMN), which serve as electron carriers in various oxidation-reduction reactions and play a pivotal role in regulating multiple pathways. In this study, our results suggested that the core targets among riboflavin, osteogenesis and osteoclastogenesis included HIF1A, HDAC2/3/4, TP53, PPARG, PPARGC1A, NFKB1, MYC and BCL2, and the potential signaling pathways of vitamin B2 involved in regulating bone health focused on HIF-1 signaling pathway, p53 signaling pathway, AGE-RAGE signaling pathway, longevity regulating pathway, and so on. HIF-1 pathway is not only responsible for the regulation of osteogenesis and osteoclastogenesis, but also for the coupling of the two processes [50–52]. Previous studies have revealed that HIF-1α can promote osteoclast activation and mediate osteogenesis via multiple steps, including epigenetic mechanisms, affecting energy metabolism, regulating angiogenesis process [50–52], etc. Given this, riboflavin could potentially exert an impact on bone homeostasis by modulating the HIF-1α pathway. This proposition can be substantiated by the evidence suggesting that flavin adenine dinucleotide (FAD) plays a role in upholding the stability of HIF-1α through the regulation of the LSD1/RACK1 pathway [53]. Furthermore, the conversion of oxidized glutathione (GSSG) to its reduced form (GSH) by glutathione reductase requires riboflavin in the FAD coenzyme form. GSH serves as endogenous antioxidant in multiple cell types to neutralize reactive oxygen species (ROS) [11]. Therefore, riboflavin deficiency will lead to the downregulation of GSH and oxidative stress, which is the predominant cause for reduced osteogenic capacities of BMSCs and enhanced osteoclastic potentials of osteoclast precursors [54, 55], finally leading to net bone loss. Inversely, sufficient riboflavin will help to produce adequate GSH and thus maintain bone homeostasis. In addition, BMSCs senescence also leads to reduced osteogenic abilities. Previously, there is study revealed that promoting cellular intake of riboflavin by upregulating expression of SLC52A1 (a riboflavin transporter, also known as GPR172B/RFVT1) plays a crucial role in antisenescence via activating mitochondrial membrane potential and inhibiting the AMPK-p53 pathway [56]. Therefore, riboflavin may also alleviate bone loss via rejuvenating BMSCs. Finally, the advanced glycation end products-receptor (AGE-RAGE) signaling pathway is involved in multiple downstream processes, such as angiogenesis, inflammation, cellular proliferation, osteogenesis, and so on. Our results indicated that riboflavin may affect the activation of AGE-RAGE pathway and thus improve bone health. Previous studies have elucidated that AGE-RAGE pathway can affect bone homeostasis in multiple links, including biomineralization [57], osteogenesis [58], osteoclastic differentiation [59], etc. Therefore, the effects of riboflavin on bone metabolism may from various respects. Taken all the facts together, riboflavin may affect bone health in a rather complexed manner, with the potential primary mechanisms focused on maintaining glutathione cycle, reducing oxidative stress, coordinating the osteogenic and osteoclastic process, and so on.

Our results indicated that the relationship of dietary riboflavin intake with osteoporosis and BMD were mediated by serum ALP. Vitamin B2 intake was found to be significantly and negatively associated with serum ALP levels, while serum ALP levels were also negatively correlated with BMD in different femoral regions and positively associated with osteoporosis. These findings were consistent with the results from previous studies. Previously, serum levels of ALP were reported to be inversely correlated with BMD levels in different anatomic sites and populations, including pelvic BMD in adults (20–59 years) [60], lumbar BMD in young adults [61], calcaneus BMD [62], hip and total body BMD in postmenopausal osteoporotic women [63], etc. ALP, an indicator of the maturation phase of the bone matrix, is an extracellular enzyme produced by osteoblasts, whose production and maturation are intricately linked to the normal growth and development of skeletal tissue [64]. When bone mineralization is compromised, osteoblasts produce an excessive quantity of alkaline phosphatase, resulting in elevated levels of ALP. Therefore, increased ALP levels are indicative of high-turnover metabolic bone diseases, including high-turnover osteoporosis [65]. Therefore, ALP levels were the indicator of bone turnover rate, and increased ALP levels indicated less bone mineralization, or more fragility and increased susceptibility to fragile fractures. In this study, our results observed a negative association between vitamin B2 intake and ALP levels, suggesting that vitamin B2 may improve bone health via reducing bone turnover rates. Such speculation is in line with the results from network pharmacological analyses, within which vitamin B2 was predicted to affect several pathways involved in suppressing osteoclastic activity and promoting osteogenic activity.

Several strengths exist in our study. Firstly, our study was performed based on a large and nationally representative population-based survey which provided adequate sample size for statistical analysis. Secondly, our study not only investigated the associations of dietary vitamin B2 intake with osteoporosis and BMD in different femoral regions, but also explored the mediation effects of serum ALP, as well as the potential targets and mechanisms regarding vitamin B2 involved in osteogenesis and osteoclastogenesis via network pharmacological analysis. Meanwhile, some limitations in the current study should also be considered. Firstly, the assessment of dietary vitamin B2 intake was based on two 24-hour dietary recall interviews, which may introduce potential limitations, such as susceptibility to recall bias, inability to evaluate the long-term association of vitamin B2 intake with osteoporosis and BMD, and so on. Secondly, the existence of residual confounding factors cannot be completely excluded despite the adjustment of potential confounding factors. In addition, some pivotal bone turnover biomarkers are not included in the NHANES database, such as procollagen type 1 N-terminal propeptide, osteocalcin, carboxy-terminal cross-linked telopeptide of type 1 collagen, amino-terminal cross-linked telopeptide of type 1 collagen, and so on, which impedes us to perform more detailed and specific analyses. Finally, the characteristics of a cross-sectional study limit its ability to establish causal relationships. As such, additional clinical intervention researches in different populations and regions are necessary to verify these findings and expand the general applicability of this study.

## Conclusion

Our study reveals a negative association between dietary vitamin B2 intake and risk of osteoporosis, as well as positive associations between dietary vitamin B2 intake and femur BMD in different regions among the US adult and female population. Moreover, serum ALP is observed to mediate the association of dietary vitamin B2 intake with osteoporosis and femur BMD. Additionally, vitamin B2 may improve bone health via modulating multiple pathways, including HIF-1 signaling pathway, longevity regulating pathway, p53 signaling pathway, AGE-RAGE signaling pathway, etc. More preclinical and clinical studies are warranted to further verify the findings from our study in the future.

## Electronic supplementary material

Below is the link to the electronic supplementary material.

Supplementary Material 1

## Data Availability

Publicly available datasets were analyzed in this study. This data can be accessible at: https://www.cdc.gov/nchs/nhanes/index.htm.

## Abbreviations

ADA: American Diabetes Association
ALP: Alkaline phosphatase
BMC: Bone mineral content
BMD: Bone mineral density
CDC: Centers for Disease Control and Prevention
DAVID: Database for Annotation, Visualization and Integrated Discovery
DXA: Dual-energy X-ray absorptiometry
FAD: Flavin adenine dinucleotide
FMN: Flavin mononucleotide
GO: Gene Ontology
GSH: Reduced glutathione
GSSG: Oxidized glutathione
KEGG: Kyoto Encyclopedia of Genes and Genomes
MEC: Mobile Examination Center
MET: Metabolic equivalent task
NHANES: National Health and Nutrition Examination Survey
PA: Physical activity
PPIs: Protein-protein interactions
RCS: Restricted cubic spline
