# Supplementary material for: Dietary riboflavin (vitamin B2) intake and osteoporosis in U.S. female adults: unveiling of association and exploration of potential molecular mechanisms
Source: Nutr J. 2025 Apr 7;24:53. doi: 10.1186/s12937-025-01103-x (PMC11974234; doi:10.1186/s12937-025-01103-x)
Supplement: Supplementary file 1 — Supplementary Material 1 [file 12937_2025_1103_MOESM1_ESM.docx]

**This file includes:**

**Table S1.** Criteria for the diagnosis of osteoporosis.

**Table S2.** Comparison of BMD in different femur regions, hip fracture and vitamin B2 intake between osteoporosis and non-osteoporosis individuals.

**Table S3.** Association between dietary vitamin B2 intake and femur BMD levels in different subgroups.

**Fig. S1.** Violin plot of dietary Vitamin B2 intake distribution in non-osteoporosis and osteoporosis participants.

**Fig. S2.** The UpSet plot, Venn plot and detailed list of the core target genes between vitamin B2 related targets, osteogenesis-related targets and osteoclastogenesis-related targets.

**Table S1.** Criteria for the diagnosis of osteoporosis.

|  | **BMD cut-off values for (g/cm^3^)** | | |
| --- | --- | --- | --- |
|  | **Osteoporosis** | **Osteopenia** | **Normal** |
| **Male** |  |  |  |
| Femur neck | ≤ 0.5875 | 0.5875-0.793 | ≥ 0.793 |
| Trochanter | ≤ 0.485 | 0.485-0.662 | ≥0.662 |
| Intertrochanter | ≤ 0.780 | 0.78-1.038 | ≥1.038 |
| Total Femur | ≤ 0.680 | 0.68-0.896 | ≥0.896 |
| Total lumbar spine | ≤ 0.782 | 0.782-0.947 | ≥0.947 |
|  |  |  |  |
| **Female** |  |  |  |
| Femur neck | ≤ 0.560 | 0.560-0.740 | ≥0.740 |
| Trochanter | ≤ 0.4625 | 0.4625-0.611 | ≥0.611 |
| Intertrochanter | ≤ 0.735 | 0.735-0.948 | ≥0.948 |
| Total Femur | ≤ 0.635 | 0.635-0.818 | ≥0.818 |
| Total lumbar spine | ≤ 0.799 | 0.799-0.958 | ≥0.958 |

**Table S2.** Comparison of BMD in different femur regions, hip fracture and vitamin B2 intake between osteoporosis and non-osteoporosis individuals.

| **Characteristics** | **Overall （n=4241）** | **Non-osteoporosis（n=2966）** | **Osteoporosis（n=1275）** | **P-value** |
| --- | --- | --- | --- | --- |
| Total Femur BMD | 0.964 (0.860-1.069) | 1.004 (0.924-1.096) | 0.768 (0.717-0.805) | <0.001 |
| Femoral Neck BMD | 0.817 (0.719-0.921) | 0.854 (0.778-0.947) | 0.646 (0.594-0.695) | <0.001 |
| Trochanter BMD | 0.720 (0.637-0.810) | 0.754 (0.688-0.832) | 0.567 (0.524-0.600) | <0.001 |
| Intertrochanter BMD | 1.144 (1.022-1.265) | 1.190 (1.096-1.299) | 0.911 (0.849-0.966) | <0.001 |
| Vitamin B2 (mg) | 1.861 (1.299-2.578) | 1.916 (1.324-2.647) | 1.694 (1.198-2.296) | <0.001 |
| Hip Fracture (N, %) |  |  |  | <0.001 |
| No | 8342 (98.687%) | 6703 (99.069%) | 1639 (97.155%) |  |
| Yes | 111 (1.313%) | 63 (0.931%) | 48 (2.845%) |  |

***Abbreviations:*** BMD, bone mineral density.

**Table S3.** Association between dietary vitamin B2 intake and femur BMD levels in different subgroups.

| **Characteristics** | **Total femur BMD** | **P interaction** | **Femoral neck BMD** | **P interaction** | **Trochanter BMD** | **P interaction** | **Intertrochanter BMD** | **P interaction** |
| --- | --- | --- | --- | --- | --- | --- | --- | --- |
| **Age** |  | 0.6943 |  | 0.782 |  | 0.2586 |  | 0.9091 |
| <=30 | 0.007 (-0.002, 0.015) 0.1172 |  | 0.006 (-0.003, 0.014) 0.1822 |  | 0.006 (-0.002, 0.013) 0.1296 |  | 0.008 (-0.002, 0.017) 0.1286 |  |
| 30-50 | 0.010 (0.004, 0.016) 0.0006 |  | 0.006 (0.001, 0.012) 0.0303 |  | 0.011 (0.006, 0.016) <0.0001 |  | 0.011 (0.004, 0.018) 0.0031 |  |
| >=50 | 0.007 (0.001, 0.013) 0.0183 |  | 0.005 (-0.000, 0.011) 0.0633 |  | 0.005 (-0.001, 0.010) 0.0850 |  | 0.009 (0.002, 0.017) 0.0135 |  |
| **BMI** |  | 0.5352 |  | 0.4308 |  | 0.0679 |  | 0.8428 |
| <=25 | 0.006 (0.000, 0.012) 0.0433 |  | 0.002 (-0.003, 0.008) 0.4010 |  | 0.004 (-0.001, 0.009) 0.0839 |  | 0.008 (0.001, 0.015) 0.0248 |  |
| 25-30 | 0.006 (-0.000, 0.013) 0.0685 |  | 0.005 (-0.002, 0.011) 0.1717 |  | 0.004 (-0.002, 0.010) 0.1478 |  | 0.007 (-0.001, 0.016) 0.0825 |  |
| >=30 | 0.014 (0.006, 0.021) 0.0004 |  | 0.011 (0.004, 0.019) 0.0029 |  | 0.014 (0.008, 0.021) <0.0001 |  | 0.014 (0.005, 0.023) 0.0027 |  |
| **Total MET** |  | 0.3725 |  | 0.3954 |  | 0.1193 |  | 0.7032 |
| Inactive | 0.007 (-0.002, 0.015) 0.1109 |  | 0.005 (-0.002, 0.013) 0.1759 |  | 0.005 (-0.002, 0.012) 0.1808 |  | 0.008 (-0.002, 0.018) 0.1021 |  |
| Insufficiently active | 0.018 (0.008, 0.027) 0.0004 |  | 0.014 (0.004, 0.023) 0.0040 |  | 0.018 (0.010, 0.027) <0.0001 |  | 0.018 (0.006, 0.029) 0.0033 |  |
| Moderate active | 0.003 (-0.009, 0.016) 0.6113 |  | 0.002 (-0.010, 0.014) 0.7469 |  | 0.005 (-0.005, 0.016) 0.3403 |  | 0.003 (-0.012, 0.018) 0.7097 |  |
| Highly active | 0.007 (0.002, 0.012) 0.0078 |  | 0.004 (-0.001, 0.009) 0.1573 |  | 0.005 (0.001, 0.010) 0.0169 |  | 0.008 (0.002, 0.015) 0.0073 |  |
| **Race** |  | 0.0621 |  | 0.4123 |  | 0.6241 |  | 0.0683 |
| Mexican American | 0.004 (-0.005, 0.014) 0.3841 |  | 0.004 (-0.005, 0.013) 0.3409 |  | 0.004 (-0.004, 0.013) 0.3387 |  | 0.004 (-0.008, 0.015) 0.5271 |  |
| Other Hispanic | 0.007 (-0.004, 0.019) 0.2166 |  | 0.004 (-0.007, 0.015) 0.4514 |  | 0.005 (-0.005, 0.016) 0.3062 |  | 0.008 (-0.005, 0.022) 0.2320 |  |
| Non-Hispanic White | 0.013 (0.008, 0.018) <0.0001 |  | 0.008 (0.003, 0.013) 0.0011 |  | 0.012 (0.007, 0.016) <0.0001 |  | 0.015 (0.009, 0.022) <0.0001 |  |
| Non-Hispanic Black | 0.001 (-0.009, 0.011) 0.8455 |  | 0.001 (-0.009, 0.011) 0.7977 |  | 0.000 (-0.008, 0.009) 0.9146 |  | 0.001 (-0.011, 0.013) 0.8481 |  |
| Other Race | 0.004 (-0.012, 0.019) 0.6670 |  | 0.005 (-0.010, 0.021) 0.4889 |  | 0.006 (-0.007, 0.019) 0.3868 |  | 0.003 (-0.017, 0.022) 0.7918 |  |
| **Education** |  | **0.0413** |  | 0.2219 |  | 0.1041 |  | **0.0108** |
| Less than high school | 0.004 (-0.004, 0.012) 0.3112 |  | 0.005 (-0.002, 0.012) 0.1954 |  | 0.005 (-0.002, 0.011) 0.1724 |  | 0.003 (-0.006, 0.012) 0.5636 |  |
| High school or equivalent | **0.014 (0.006, 0.022) 0.0004** |  | 0.011 (0.004, 0.019) 0.0041 |  | 0.012 (0.005, 0.019) 0.0007 |  | **0.019 (0.009, 0.028) 0.0001** |  |
| Some college or above | 0.008 (0.003, 0.013) 0.0025 |  | 0.004 (-0.001, 0.009) 0.1192 |  | 0.007 (0.002, 0.012) 0.0028 |  | 0.009 (0.002, 0.015) 0.0066 |  |
| **Marital Status** |  | 0.5599 |  | 0.804 |  | 0.7903 |  | 0.4473 |
| Married/living with partner | 0.008 (0.003, 0.013) 0.0028 |  | 0.006 (0.001, 0.011) 0.0147 |  | 0.007 (0.003, 0.012) 0.0013 |  | 0.008 (0.002, 0.014) 0.0085 |  |
| Never married | 0.005 (-0.004, 0.014) 0.2623 |  | 0.002 (-0.006, 0.011) 0.5951 |  | 0.005 (-0.002, 0.013) 0.1576 |  | 0.005 (-0.005, 0.015) 0.3313 |  |
| Others | 0.013 (0.005, 0.020) 0.0015 |  | 0.008 (0.001, 0.015) 0.0214 |  | 0.009 (0.003, 0.016) 0.0061 |  | 0.015 (0.006, 0.025) 0.0013 |  |
| **Menopause** |  | 0.2357 |  | 0.6621 |  | 0.1129 |  | 0.3003 |
| No | 0.009 (0.005, 0.014) 0.0002 |  | 0.005 (0.000, 0.010) 0.0315 |  | 0.009 (0.005, 0.013) <0.0001 |  | 0.011 (0.005, 0.016) 0.0003 |  |
| Yes | 0.007 (0.001, 0.013) 0.0192 |  | 0.006 (0.001, 0.012) 0.0242 |  | 0.005 (-0.000, 0.010) 0.0535 |  | 0.008 (0.001, 0.015) 0.0257 |  |
| **Prednisone or cortisone intake** |  | 0.8993 |  | 0.8404 |  | 0.7377 |  | 0.7652 |
| Yes | 0.011 (-0.006, 0.028) 0.2100 |  | 0.009 (-0.008, 0.025) 0.3111 |  | 0.012 (-0.002, 0.027) 0.0972 |  | 0.009 (-0.011, 0.029) 0.3886 |  |
| No | 0.008 (0.004, 0.012) <0.0001 |  | 0.006 (0.002, 0.009) 0.0030 |  | 0.007 (0.004, 0.010) <0.0001 |  | 0.010 (0.005, 0.014) <0.0001 |  |
| **Milk consumption** |  | 0.0732 |  | 0.372 |  | **0.0405** |  | 0.0793 |
| Never/rarely (never or less than once a week) | 0.007 (-0.000, 0.014) 0.0583 |  | 0.006 (-0.001, 0.013) 0.1033 |  | 0.006 (-0.001, 0.012) 0.0749 |  | 0.007 (-0.002, 0.015) 0.1329 |  |
| Sometimes (once or more a week but less than once a day) | 0.002 (-0.006, 0.010) 0.6593 |  | 0.002 (-0.006, 0.010) 0.6228 |  | 0.001 (-0.006, 0.008) 0.7766 |  | 0.003 (-0.007, 0.013) 0.5862 |  |
| Often (once a day or more) | 0.013 (0.007, 0.018) <0.0001 |  | 0.008 (0.003, 0.013) 0.0021 |  | **0.011 (0.007, 0.016) <0.0001** |  | 0.015 (0.008, 0.021) <0.0001 |  |
| **Diabetes** |  | 0.1984 |  | 0.2996 |  | 0.1041 |  | 0.2066 |
| Yes | 0.015 (0.001, 0.030) 0.0360 |  | 0.012 (-0.002, 0.026) 0.0851 |  | 0.015 (0.002, 0.027) 0.0209 |  | 0.018 (0.001, 0.035) 0.0443 |  |
| No | 0.008 (0.004, 0.012) 0.0001 |  | 0.005 (0.002, 0.009) 0.0059 |  | 0.007 (0.003, 0.010) 0.0001 |  | 0.009 (0.004, 0.014) 0.0003 |  |
| **Smoking** |  | 0.0631 |  | 0.1157 |  | 0.1305 |  | **0.0464** |
| No | 0.008 (-0.001, 0.017) 0.0849 |  | 0.011 (0.002, 0.019) 0.0116 |  | 0.009 (0.001, 0.017) 0.0239 |  | 0.006 (-0.005, 0.017) 0.3029 |  |
| Yes | 0.002 (-0.005, 0.008) 0.6486 |  | 0.000 (-0.006, 0.007) 0.8850 |  | 0.003 (-0.003, 0.009) 0.3584 |  | 0.001 (-0.007, 0.010) 0.7474 |  |
| Missing | 0.012 (0.007, 0.017) <0.0001 |  | 0.007 (0.002, 0.012) 0.0055 |  | 0.009 (0.005, 0.014) <0.0001 |  | **0.015 (0.009, 0.021) <0.0001** |  |

***Abbreviations:*** BMI, body mass index; MET, metabolic equivalent task; PIR, ratio of family income to poverty; ALP, Alkaline phosphatase, OR, odds ratio; CI, confidence interval. The variables adjusted for subgroup analyses were consistent with Model 3 in Table 2 except the stratifying variable.


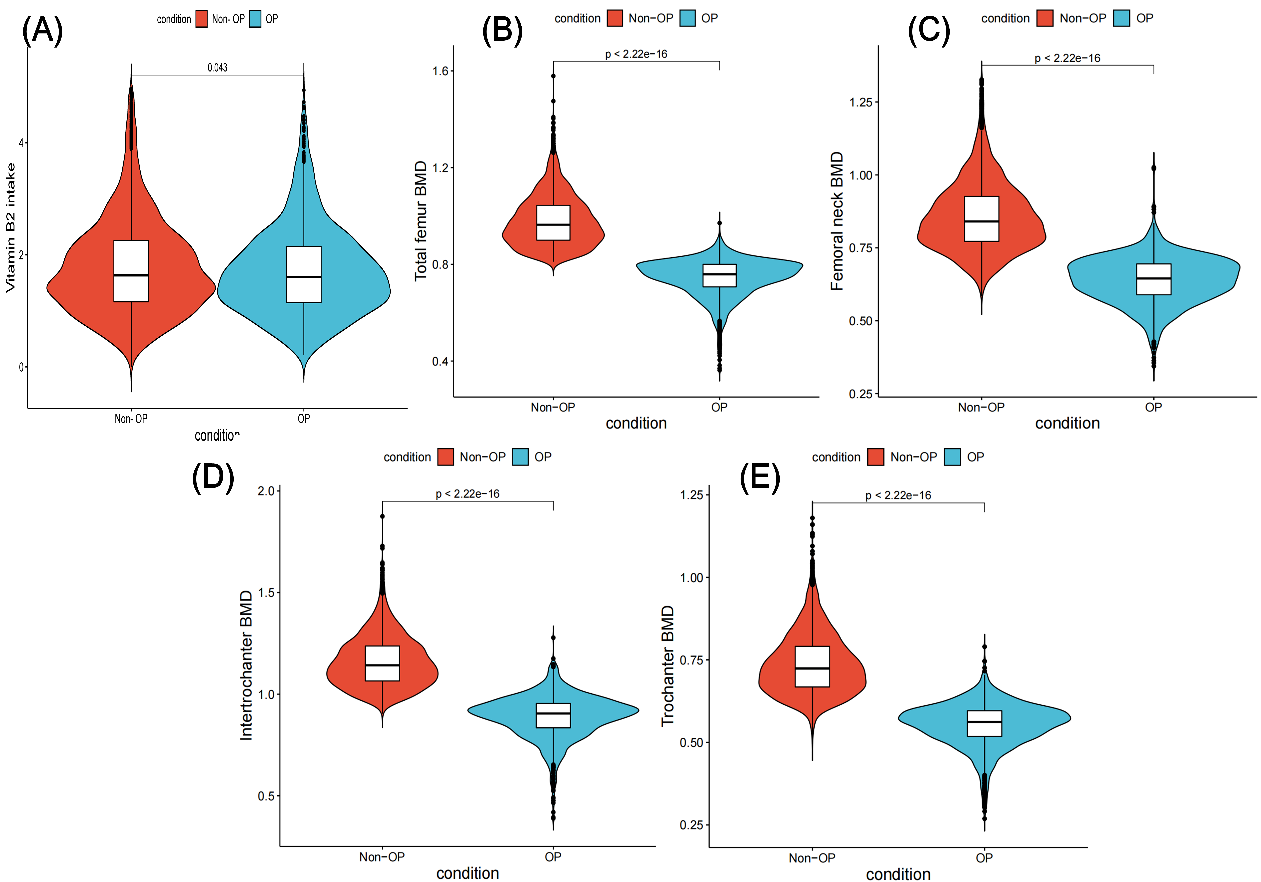
**Fig. S1.** Violin plot of showing the distribution differences in dietary Vitamin B2 intake, total femur BMD, trochanter BMD, intertrochanter BMD, and femoral neck BMD between non-osteoporosis and osteoporosis participants. (A) dietary Vitamin B2 intake; (B) total femur BMD; (C) femoral neck BMD; (D)intertrochanter BMD; (E) trochanter BMD.


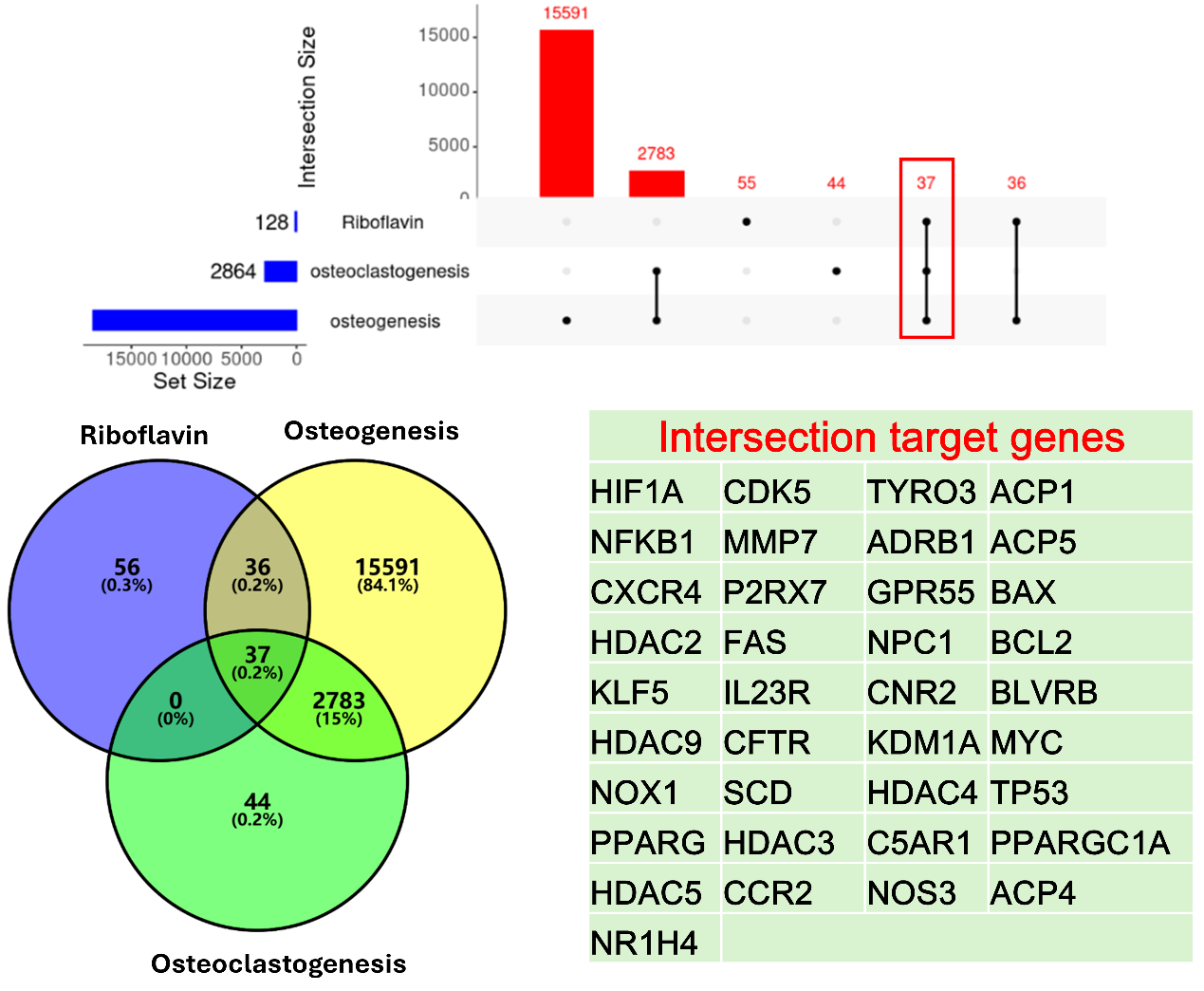


**Fig. S2.** The UpSet plot, Venn plot and detailed list of the core target genes between vitamin B2 related targets, osteogenesis-related targets and osteoclastogenesis-related targets.
